# The genome of the arapaima (*Arapaima gigas*) provides insights into gigantism, fast growth and chromosomal sex determination system

**DOI:** 10.1038/s41598-019-41457-x

**Published:** 2019-03-28

**Authors:** Kang Du, Sven Wuertz, Mateus Adolfi, Susanne Kneitz, Matthias Stöck, Marcos Oliveira, Rafael Nóbrega, Jenny Ormanns, Werner Kloas, Romain Feron, Christophe Klopp, Hugues Parrinello, Laurent Journot, Shunping He, John Postlethwait, Axel Meyer, Yann Guiguen, Manfred Schartl

**Affiliations:** 1 University of Wuerzburg, Physiological Chemistry, Biocenter, 97074 Wuerzburg, Germany; 2 Key Laboratory of Aquatic Biodiversity and Conservation of the Chinese Academy of Sciences, Institute of Hydrobiology, Chinese Academy of Sciences, Wuhan, Hubei 430072 China; 3 University of Chinese Academy of Sciences, Beijing, 100049 China; 4 Leibniz-Institute of Freshwater Ecology and Inland Fisheries, IGB, Müggelseedamm 301, D-12587 Berlin, Germany; 5 Reproductive and Molecular Biology Group, Departament of Morphology, Institute of Biosciences, UNESP, Botucatu, Brazil; 6 INRA, UR1037 LPGP, Fish Physiology and Genomics, F-35042 Rennes, France; 7 MIAT INRA Toulouse, CS 52627, 31326 Castanet-Tolosan, France; 8 Montpellier GenomiX (MGX), c/o Institut de Génomique Fonctionnelle, 141 rue de la cardonille, 34094 Montpellier Cedex 05, France; 9 Institute of Neuroscience, University of Oregon, Eugene, Oregon, OR 97401 USA; 10 Lehrstuhl für Zoologie und Evolutionsbiologie, Department of Biology, University of Konstanz, Universitätstraße 10, 78457 Konstanz, Germany; 11 Comprehensive Cancer Center Mainfranken, University Hospital, 97080 Würzburg, Germany; 12 Hagler Institute for Advanced Study and Department of Biology, Texas A&M University, College Station, Texas 77843 USA

## Abstract

We have sequenced the genome of the largest freshwater fish species of the world, the arapaima. Analysis of gene family dynamics and signatures of positive selection identified genes involved in the specific adaptations and unique features of this iconic species, in particular it’s large size and fast growth. Genome sequences from both sexes combined with RAD-tag analyses from other males and females led to the isolation of male-specific scaffolds and supports an XY sex determination system in arapaima. Whole transcriptome sequencing showed that the product of the gland-like secretory organ on the head surface of males and females may not only provide nutritional fluid for sex-unbiased parental care, but that the organ itself has a more specific function in males, which engage more in parental care.

## Introduction

The Amazonian freshwater fish arapaima (*Arapaima gigas*) has a most remarkable biology. This legendary fish can reach a body length of almost 3 meters placing it the largest freshwater fish, exhibits the fastest known growth rates, and – supporting its importance for aquaculture – has the best food conversion so far recorded in fish^1,2^. Known as pirarucu in Brazil and paiche in Peru, it belongs to the bonytongues (Order Osteoglossiformes), one of the most basally diverging lineages of the teleost fish. Its natural distribution covers most of the Amazon River basin in Peru and Brazil, and it has been introduced as an aquaculture species to other rivers in tropical South America. As an obligate air-breathing fish obtaining up to 95% of its oxygen uptake by breathing, it is able to tolerate extremely low oxygen levels in the water, and is less susceptible to ammonia or nitrite intoxication due to its degenerated gills.

The combination of unusual adaptations make arapaima a promising candidate for aquaculture. But so far, it has not been established widely in aquaculture, partially due to deficits in knowledge about its sexual development, allowing controlled reproduction in captivity, and lack of information about the molecular and biochemical mechanisms involved in its fast and gigantic growth^3^. So far, genomic resources are sparse^4^ and far below what is available for other important aquaculture fish. In South America arapaima is a high-priced fish for commercial fisheries. Sadly, the drastic decline in natural stocks and genetic bottlenecks^5^ brought arapaima towards the brink of extinction^6^ and illicit poaching - despite its status of protection - continues to threaten remaining populations^7,8^. Destruction of its natural habitats through various causes is also contributing to the immanent threat of extinction of this iconic species.

Gigantism and muscle growth separate arapaima (>2.5 m, >100 kg) from its relative, the much smaller Asian arowana *Scleropages formosus*^9^. During the first year, juvenile arapaima exhibits the fastest growth rates recorded in fish, reaching weights between 10 to 15 kg at an extraordinary efficient food conversion rate (FCR < 0.7). Genome wide studies on this phenomenon are not available but are desirable for understanding this spectacular growth ability and they will allow comparisons with the *S*. *formosus* genome, both in ontogenetic and evolutionary contexts.

Sex determination and the regulation of sexual maturity onset (“puberty”) are still largely unknown for arapaima^10^ because occasional reproduction in ponds so far has not provided enough information to establish captive breeding and aquaculture^11^. Arapaima is a fractional spawner and maturation of the ovary is synchronous^12^. Only the left gonad is functional and the right one is atrophied, both in males and females^12,13^. Therefore, biopsies via the gonoduct are difficult to take and, with regard to the body size, stressful as well as labour intensive. Here, a sex marker would facilitate reproductive management substantially^14^.

A morphological specialization in relation to reproduction is the so-called “secretory organ”, which has been reported to function in parental care^15^. Present on the head of males and females, it shows no sex-specific morphological differences. During the reproductive period, the secretory organ secretes a milky fluid that is thought to provide nutrients to the fry^16^. Due to a relatively low protein content of the secretion further functions such as pheromonal gland have been discussed. As such, agglomeration pheromones for the juveniles as well as priming pheromones targeting the opposite sex can be hypothesized. However, males engage much more in parental care and stay with the offspring for up to 3 months, while females leave the father and the offspring after about one month to reproduce again with other males during the same season. Males guide the offspring swimming above his darkened head (which appears to provide camouflage) to nutrient-enriched feeding areas. Insights into the reproductive biology of arapaima will not only support the development of reproductive technologies in aquaculture but will also contribute a new understanding of the evolution of sex determination and sex differentiation in fish.

The availability of extended genomic resources^4^, including several genomes and a wide spectrum of transcriptomes will be highly useful for a better understanding of not only of the biology of this spectacular fish, but will provide necessary information for establishing arapaima in recirculation aquaculture facilities and for supporting conservation measures and, importantly, the restoration of natural stocks. Here, we focused on the sex determination system and the function of the secretory field, hypothesizing a sex-specific function as pheromonal gland.

## Results and Discussion

### Genome assembly and annotation

We used a whole genome shotgun approach with Illumina technology (Hiseq2500) to sequence the genome of one male and one female at 58.6 and 59.8x coverage, respectively (Fig. S1). The DNA was extracted from fin tissues, and the libraries were produced using the Truseq DNA Nano sample preparation kit. The DISCOVAR (version 52488) assembly resulted in 52,688 scaffolds with size ranging from 138 bp to 2,146 kb for the female (average size 13 kb, N50 315 kb); for male, 60,055 scaffolds with size ranging from 192 bp to 3,323 kb (average size 12 kb, N50 285 kb). The total size of the male assembly is 666 Mb and of the female 664 Mb compared to the estimated 790 Mb from cytometric C-value measurements^17^.

Based on the Benchmarking Universal Single-Copy Orthologs (BUSCO) method from the vertebrate database^18^, the completeness of the male assembly is estimated to being 96.1% and in that of the female 95.7% (Table S1). Because of its higher contiguity, we chose the male assembly for annotation. With our own in-house assembly pipeline (Fig. S1), we predicted 26,755 genes, out of which 21,701 (81.1%) were identified with known Pfam protein domains. BUSCO analysis revealed 2,471 (95.5%) out of 2,586 conserved vertebrate genes to be annotated and complete (Table S1).

**Figure 1:**
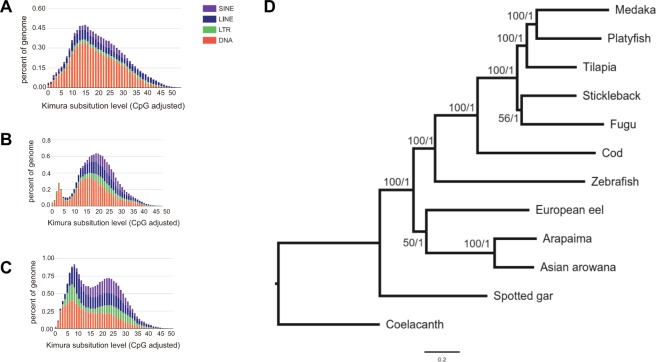
Interspersed repeat landscape for arapaima, Asian arowana and European eel and phylogenetic tree. (**A**–**C**) The interspersed repeat landscape, revealing the copy-divergence analysis of TE classes, based on Kimura distances. Percentages of TEs in genomes (Y-axis) are clustered based on their Kimura values (X-axis; K-values from 0 to 50; arbitrary values). Older copies are located on the right side of the graphs while rather recent copies are located on the left side. (**A**) is for European eel, (**B**) arapaima, (**C**) Asian arowana. (**D**) The phylogenetic tree, generated using 234 one-to-one ortholog protein sequences. Values at the nodes are Maximum Likelihood bootstrap percentages/Bayesian posterior probability values.

Analysis of repeats in the male genome assembly revealed that repetitive elements constitute 16.46% of the genome, which is in the range of other teleost genomes of similar size. One category of repetitive elements, the transposable elements (TEs), are always of interest with regards to their important role in the evolution of genes, gene networks, and genomes^19^. In male arapaima, TEs account for 16.21% of the male and 16.77% of the female genome (Table S2). To investigate TE dynamics, we calculated the distribution of TEs based on Kimura Distance for European eel (Fig. 1A), arapaima (Fig. 1B) and Asian arowana, the only other sequenced osteoglossomorph genome (Fig. 1C), as an approximation of relative ages of TEs^20^. Two major transposition bursts are apparent. The older one comprises all major classes, while the more recent burst mainly affected DNA elements (Fig. 1B). After the first burst, all other families obviously contracted to the lower levels observed today. Compared to other teleosts, the Kimura profile of arapaima also follows the pattern of “generally one or two main bursts” with some significant interspecific differences^19^. Usually, if there are two bursts, they are similar with either one major class or all classes contributing in a similar way to the expansion. However, arapaima is so far unique amongst the analyzed vertebrate genomes with one burst of mainly DNA transposons and another one to which all classes of TEs contributed.

### Phylogenomics

The phylogenetic relationships between arapaima and eleven other bony fish species were determined based on 234 one-to-one orthologs. We used Maximum Likelihood and Bayes methods, respectively, to reconstruct the phylogenetic tree. Both methods resulted in the same topology where arapaima is a sister group to the Asian arowana (Fig. 1D). The topology of the tree is in perfect agreement with the current fish tree of life and other phylogenomic studies^4,21,22^. According to divergence time estimations using MCMCTree, the two bony tongue lineages split approximately 138.4 million years ago (Fig. S2). This is about 50 million years older than a previous calculation^4^ but still 20–30 millions years younger than the estimate from previous studies based on fossil evidence and the Afro-South American drift and from molecular phylogeny based on mitochondrial DNA sequences^23,24^. This may be indicative of a slower than average nuclear evolutionary rate in this lineage^25^.

### Gene family dynamics

Lineage and species adaptations can lead to variations in the sizes of gene families. In the arapaima genome, we identified nine gene families that have undergone significant expansion during evolution, and 21 gene families that noticeably contracted (Table S3). Intriguingly, gene families related to immunity (immunoglobulin light/heavy, major histocompatibility complex class I & II, elastase, granzyme, tissue factor pathway inhibitor and novel immune-type receptor) and odorant receptor family E were found to be contracted, while odorant receptor, family F and H, expanded. In particular, the elastase family is very small in European eel, arapaima and Asian arowana (0, 0, 2 members) while it has many members (>10 to >40) in most other fish. The family ‘novel immune-type receptor’ is also small in arapaima, arowana and European eel (2, 7, 1), but large in other fish (12–79). The small size of these immune response-related gene families among Elopomorpha and Osteoglossomorpha but a higher number in spotted gar (20) may indicate that the basal teleost groups have lost such genes and thus have a less elaborate gene repertoire for this trait.

To investigate the dynamics of gene families related to olfaction, we adapted the identification method and classification from Niimura^26^ and performed a comparative analysis. In total, we identified 1,645 functional odorant receptors (OR), 103 functional trace amine-associated receptors (TAAR) and 107 vomeronasal receptors (VR) from 18 vertebrates (Table S4). Among those ORs, the α and γ-ORs sense air-borne odors^26^. We found quite a number of 〈 and ©-ORs in spotted gar and coelacanth that are absent or scarce in other fish, including arapaima, but abundant in tetrapods. In coelacanth the presence of the 〈 and © receptors may have facilitated the evolution of the water-to-land transition in the basal sarcopterygians. Their abundance in the gar genome indicates that there has been a reduction up to total loss in some species in the teleost lineage. Our results also revealed a larger repertoire of water-soluble and air-borne/water odorant receptors in herbivorous than in carnivorous teleosts, in agreement with a previous study^27^. One possible hypothesis is that plant eaters need more odorant information to smell whether a potential food is toxic to them or not. For the ORs that receive water-borne odors, arapaima has a similarly low number of δ genes as arowana, but the highest number of η ORs of all genomes analyzed.

Because arapaima is predominantly carnivorous, we also investigated the gene family of taste receptors, an important factor in the development of carnivore habits. *tas1r1*, the receptor gene for sensing umami, the taste of meat, which lost function by a frameshift mutation in giant panda when diet changed from carnivory to herbivory^28^, is duplicated in the arapaima genome (g19850.t1 and g20353.t1), while *tas1r*2, the receptor gene for sensing sweet, was lost. This is in accordance with adaptation to a diet that is mainly composed of prey and does not contain much saccharides.

### Genes under selection

Positively selected genes (PSG) are the result of adaptive evolution and often associated with new enhanced or selected functions of an organism^29^. Such genes may be recognized by dN/dS ratios >1. To identify PSGs characteristic of the arapaima lineage, we first screened 3,128 one-to-one orthologs that were retrieved from at least 8 of the 12 fish species shown in Fig. 1D and tested for signs of positive selection in five lineages (arapaima, Asian arowana, European eel and the two most recent common ancestor lineages; branch-site model in codeML). Then, from the 226 arapaima PSGs (FDR-adjusted-p < 0.01) we removed those also showing signs of positive selection (FDR-adjusted-p < 0.05) in other lineages, and retrieved 105 arapaima exclusive PSGs (Table S5). This list contained genes related to bone metabolism (Osteoclast Stimulating Factor 1, *ostf*1), cell growth, and cell division (Cyclin C, *ccnc*; Cell Division Cycle 5 Like, *cdc5l*). GO enrichment of these genes revealed “cyclin-dependent protein kinase holoenzyme complex” to be enriched (Table S6), which may indicate a relation between cell cycle control and body size in the context of the gigantism as an important trait in arapaima.

To expand the search grid, we also included 12,929 one-to-one orthologs between arapaima and Asian arowana to test their pairwise dN/dS value, and retrieved 159 genes with pairwise dN/dS > 1 ((FDR-adjusted-p < 0.01)) that had no sign of positive selection in the lineage of Asian arowana, European eel and the two most recent common ancestor (from the above branch-site model test, Table S7). These genes include again several known to be involved in growth and cell proliferation (Proto-oncogene tyrosine-protein kinase Src, *src*; Bone morphogenetic protein 7, *bmp7*; Growth Arrest Specific 2, *gas*2; Runt Related Transcription Factor 2, *runx*2; Neuron Navigator 2, *nav2* and Centrosomal Protein 295, *cep295*). GO term analysis on these genes showed that functions related to development, in particular, development of the musculo-skeletal system, are enriched in positively selected genes (Table S8), suggesting that those genes contribute to the large body size of arapaima. This finding is particularly interesting because arapaima does not possess intermuscular bones (ray-like free bone near the skin, laterally in the body) as other related fish species and thus relies for coordination of movement more on the support provided by the interaction of muscle and vertebrae.

Beside PSGs, genes showing substantial different dN/dS values between the arapaima and Asian arowana lineages are of interest, because Asian arowana is the closest known species but has considerable difference in phenotype and life history from arapaima. Thus, 5,882 one-to-one orthologs of arapaima, Asian arowana and European eel were ranked according to *|log*_*2*_*(ω*_1_*/ω*_2_*)|* value (ω_1_ refers to arapaima-European eel pairwise dN/dS value and ω_2_ refers to Asian arowana-European eel pairwise dN/dS value). The top 5% (295) genes of the ranked list were selected, among which 118 genes showed higher arapaima-European eel pairwise dN/dS values and no sign of positive selection in the lineages of Asian arowana, European eel and the two most recent common ancestor (from the above branch-site model test, Table S9). This analysis also identified genes related to cell growth and division (Interleukin 1 Receptor Associated Kinase 1 Binding Protein 1, *irak1bp1*; IGF Like Family Receptor 1, *igflr1*; *ccnc* and Cyclin Dependent Kinase 20, *cdk20*). Among these genes, functions related to cell cycle are also enriched (Table S10).

**Figure 2:**
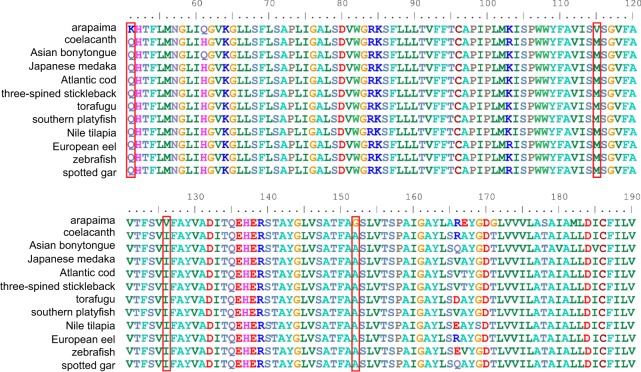
Multiple Protein Sequence Alignments of Mfsd14a (Hiat1) for *L*. *oculatus* (spotted gar), *O*. *latipes* (Japanese medaka), *X*. *maculatus* (southern platyfish), *O*. *niloticus* (Nile tilapia), *G*. *aculeatus* (three-spined stickleback), *T*. *rubripes* (torafugu), *G*. *morhua* (Atlantic cod), *D*. *rerio* (zebrafish), *L*. *chalumnae* (coelacanth), *A*. *anguilla* (European eel), *S*. *formosus* (Asian bonytongue) and *A*. *gigas* (arapaima). Sites with arapaima-unique residues surrounded by conserved sequence are marked with red frame.

In addition, besides characteristic dN/dS values in a codon-based evolution model, residues uniquely substituted in a lineage of interest were previously shown to indicate significant changes in protein function^30,31^. We screened 6,052 homologs (orthologs and paralogs) from all 12 species for arapaima-unique residues and identified 4,677 arapaima-unique residues in 1,959 protein sequences. These arapaima-unique residues were scored based on the conservation of flanking residues, and the protein sequences, based on the score of their arapaima-unique residues (see the formula in Materials and Methods). The higher the score of a residue, the more conservative the sequence region that harbors it, which gives such substitutions a higher significance. In extension, the higher the score of a protein sequence, the more unique substitutions of higher significance it harbors. Hence we ranked them respectively in a descending order, and kept the top 1% of each list (Tables S11 and S12). Again, cell division-related genes were found (Cyclin-dependent kinase 9, *cdk9* and Cyclin L1, *ccnl1*). Another top-ranked gene is *mfsd14a* (or *hiat1*, Hippocampus abundant transcript 1) (Fig. 2). Even though fish lack a tetrapod-like hippocampus structure, the pallial region of the teleost telencephalon contains subdivisions that are presumably homologous to the hippocampus in amniotes^32^, and might be involved in spatial memory and navigation in the frequently turbid habitat of the Amazon tributaries. However, the precise function of *mfsd14a* in arapaima is still waiting to be uncovered.

### Sex-specific sequences

To obtain insights in possible sex-specific sequences, a RAD-tag analysis of 25 females and 25 males was performed. From this analysis we extracted 30 RAD-tags present in most males but absent in most females (Table S13). We further extracted all contigs/scaffolds that were present only in the female or only in the male genome, which identified 254 female-specific scaffolds and 281 male-specific scaffolds, accounting for ~0.12% of each genome, much higher than that from previous study (0.01%)^4^. When those male-specific scaffolds were blasted on the Asian arowana genome 133 had a hit with e-value <1e-5 (Table S14). However, these did not form obvious synthenic groups. When the 30 RAD-tag sequences were blasted against the male and female assembly, seven RAD-tags exclusively matched to the male-specific scaffolds, 18 matched both to the male-specific scaffolds and to additional scaffolds in one or both reference genomes, and 5 matched reference genome-scaffolds that were not sex-specific. None of the 30 sex-associated RAD-tags matched only to female-specific scaffolds (Table S15). The identification of male-specific RAD-tags but no female-specific tags is compatible with an XY chromosomal sex-determination system with a reasonable molecular differentiation of the sex chromosomes. Arapaima, however, lacks heteromorphic sex chromosomes detected cytologically^33^.

Male-specific scaffolds lacked any annotated protein coding genes previously known to be related to sex determination or gonad development (Table S16). Comparing those genes to the “potential male-specific gene” from Vialle *et al*.^4^, we found *amdhd1* (probable imidazolonepropionase) and *cd48* (CD48 antigen) to be contained in both datasets. It is interesting to note that these scaffolds are considerably different from the rest of the genome with respect to TE content (Fig. S3). TEs are much more prevalent in the sex-linked scaffolds (46.1%) than in the non-sex-linked scaffolds (14.9%). The dynamics of TEs on sex-linked scaffolds is also different from the rest of the genome. They consist preponderantly of LINE elements that are derived from a recent burst (69% of LINEs with Kimura substitution <10). A higher TE content and local TE expansion due to reduced recombination in a male-specific region is a typical feature of Y-chromosomes^34^.

We hypothesized that the Y-specific region may have been derived from a duplication of an autosomal region, because several RAD-tags mapped not only to male-specific scaffolds but also to the part of the genome that is common to male and female (with slightly lower scores). Such a situation has been reported for the Y-chromosomes from several other fish species (e.g. medaka, pejerrey, rainbow trout, reviewed in^35^). We thus searched the arapaima reference genome for regions paralogous to the male-specific scaffolds that had male-specific tags using LAST^36^ and found four scaffolds that are paralogous to putative Y-linked scaffolds (Fig. S4). We then inspected these Y-scaffold paralogs for linked genes with annotation (Table S17). However, none of these genes is related to a gene that has been implicated in sex determination in other fish and would be a candidate for a master sex regulator.

In the course of sex chromosome evolution, genes that are beneficial for only one sex (e.g. spermatogenesis genes) or even detrimental to the opposite sex (sex-antagonistic genes), can become linked to the sex-determining locus. To search for such genes, we looked for sex-biased gene expression patterns in testis, ovary, male and female secretory organs. First, to compare gene expression between testis and ovary, we plotted the 20,927 genes that have RNA-seq reads mapped either in testis or/and in ovary. Among them, 26 genes that are covered by more testis reads than ovary reads are located in male-related scaffolds (male-specific scaffolds and their paralogous autosomal region) (Fig. S5).

To further identify genes that are differentially expressed between in testis and in ovary, we calculated and compared the expression level using DESeq2, those genes with read counts >500 in one gonad and unexpressed in the other, meanwhile, with |log2FC| >4 were identified as differentially expressed gene (Table S18). The same procedures were conducted for genes expressed in the male or/and female secretory organ. On the contrary, none of the differentially expressed genes mapped to one of the sex-specific scaffolds (Table S17).

Genes that are generally known to be involved in gonad functions and structure and show a sex-biased expression in other fishes, display the expected expression profile (Fig. S6).

**Figure 3:**
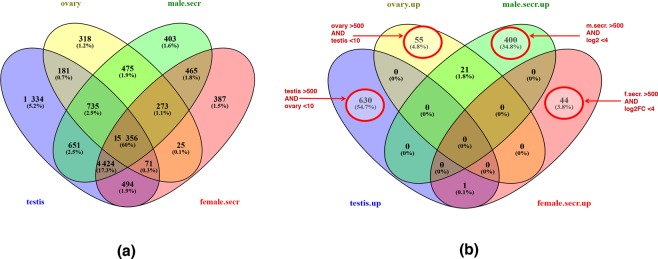
(**a**) Venn diagram of the total number of gene expressed in testis, ovary, male and female secretory. (**b**) Venn diagram of the number of genes differentially expressed in ovary vs. testis (base mean >500 AND log2FC > 10) and male vs. female secretory organ (base mean >500 AND log2FC > 4), respectively. The Venn diagram was created using Venny (http://bioinfogp.cnb.csic.es/tools/venny/).

Both male and female arapaima produce during the breeding season a fluid from their cephalic secretory organ that is released for nurturing the fry. Despite such an obvious common function postulated for male as well as female, it was surprising to find a large number (n = 466) of differentially expressed transcripts in male and female secretory organs, with 421 transcripts exclusively expressed in males but only 45 transcripts expressed only in females (Fig. 3, Tables S18 and S19). Such pronounced sex differences were not obvious on the protein level from a recent proteomic study of arapaima secretory organs^16^. Interestingly, the secretory organ genes expressed exclusively in males were enriched in several pathways, e.g. insulin signaling, glycolysis and gluconeogenesis, and ovarian infertility genes (Table S19, Fig. S1). Genes encoding the oocyte-specific growth factors Gdf9 (Growth Differentiation Factor 9) and Bmp15 (Bone morphogenetic protein 15), the folliculogenesis transcription factor Figl alpha, and several egg structure proteins, e.g. zona pellucida proteins, were highly and exclusively expressed in the male secretory organ, but not or extremely lowly expressed in female (Table S19). Whether this apparent ‘ectopic expression’ is related to a signal from the male to the accompanying female has to be evaluated by further studies. One hypothesis is that such a signal may prevent the female to enter the next reproductive cycle while paternal care of the previous brood is still ongoing. Similarly, the fact that several growth factors are released can motivate studies on a possible role of these factors for the known fast growth rate of arapaima fry.

## Conclusions

We generated a draft genome sequence of good contiguity that provided a useful reference for phylogenomic and comparative genomic evolutionary analyses. Much more work can be done with these genomic resources to improve our understanding of the biology and evolutionary ecology as well as questions connected to aquaculture, fisheries and conservation management.

To this end, we identified candidate genes that may be related to arapaima-specific traits, for instance fast growth and large body size, adaptations to a carnivorous lifestyle, and the function of the secretory organ. In this context, it was surprising to find male-specific gene expression in the secretory organ that assigns both a fry-nutrition function and also a pheromone-type signaling functioning to local females.

For the first time, we inferred from the genomic data a possible genetic sex determining system of male heterogamety in this species that presents homomorphic chromosomes for both sexes. We identified male- (Y-)specific scaffolds that can be potentially useful to identify the master male sex determining gene(s) and to design molecular markers that are highly desirable for aquaculture breeding and wild stock management.

## Materials and Methods

### Ethic statement

Fish were sacrificed by state-of-the-art humane killing (American Veterinary Medical Association, Canadian Council of Animal Care in Science). The experiments were carried out in accordance with the European Directive 2010/63/EU and German national legislation (Animal protection law, TierSchG). All experimental protocols that are part of this study were approved through an authorization (File # ZH 114, issued 06.02.2014) of the LAGeSo, Berlin, Germany.

### Genome sequencing and assembly

*A*. *gigas* DNA for Illumina shotgun sequencing was derived from fin tissue of a single adult female (ID F3) and from a single adult male (ID M14). Libraries were produced using the Truseq DNA Nano sample prep kit using the 550 pb insert size option. Libraries were sequenced on a Hiseq 2500 using rapid v2 PE 2*250 nt mode (half a lane per library). All sequences were assembled with DISCOVAR (version 52488) *de novo* ((https://software.broadinstitute.org/software/discovar/blog/)) using default parameters.

### RAD-tag sequencing and analysis of sex-specific tags

Genomic DNA was extracted from 90% ethanol-preserved fin clips using a classical phenol/chloroform protocol. The arapaima RAD-tag library was built according to standard protocols^37^, using Sbf1 as a single restriction enzyme, and sequenced on a single lane of Hiseq2500 using the v4 SR100nt mode. The resulting read file was then demultiplexed using the process_radtags.pl script of STACKS software version 1.44^38^ with default settings. Demultiplexed reads were analyzed with the denovo_map.pl STACKS script with the following settings: m = 3 (more than 3 reads are needed to build a stack of sequences), M = 0 (zero mismatch allowed when building stacks in one sample), N = 0 (zero mismatch allowed when merging stacks in the catalog),–gapped not set (gapped assembly disabled), H not set (calling haplotypes from secondary reads disabled),–max_locus_stacks = 1 (ustacks max locus per stack set to 1), and–keep_high_cov set (ustacks lumberjack step deactivated). Zero values for the M and N setting produce stacks of reads with no polymorphism, each resulting stacks of reads containing single allele reads or non polymorphic stacks of reads.

### Repeat annotation and TE analysis

The genome assembly was inspected by RepeatModeler (http://www.repeatmasker.org/RepeatModeler, version 1.0.4) to generate a library of known and model a de-novo repeats (Fig. S1). This library, together with our in-house fish-specific repeat library, was incorporated by RepeatMasker (http://www.repeatmasker.org/RMDownload.html, version open-4.0.7) to annotate and mask repeat elements from the genome assembly. For TE insertion repeats, we also used RepeatLandscape (https://github.com/caballero/RepeatLandscape) to calculate Kimura distance as a measure of age, and to display a relative age profile.

### Genome annotation

Genome annotation was done by combining gene evidence from homology annotation, *de novo* annotation and transcripts (Fig. S1). For homology evidence we downloaded 354,871 protein sequences from Ensembl species (Ensembl release 87) *H*. *sapiens* (human), *D*. *rerio* (zebrafish), *L*. *chalumnae* (coelacanth), *P*. *marinus* (sea lamprey), *T*. *rubripes* (torafugu), *T*. *nigroviridis* (spotted green pufferfish), *G*. *aculeatus* (three-spined stickleback), *O*. *latipes* (Japanese medaka), *C*. *milii* (elephant shark)^39^, and from *S*. *formosus* (Asian bonytongue)^9^, the closest known relative to arapaima. These sequences were then aligned to the repeat-masked genome sequence using exonerate2.2.0 (http://www.ebi.ac.uk/about/vertebrate-genomics/software/exonerate) and Genewise2-2-0^40^, respectively, to predict potential gene structures. To improve the efficiency of Genewise, we also used genBlastA1.0.1^41^ to roughly locate each protein on the genome sequence before Genewise was implemented. For gene evidence from *de novo* annotation, AUGUSTUS3.2.3^42^ was used to predict genes on the repeat-masked genome sequence with ‘zebrafish’ as the parameter for –species flag. In parallel, we also used GeneMark-ES^43^ in unsupervised training model (-ES) for the *de novo* prediction. For the gene evidence from transcripts, we collected RNA-seq reads from eyes, gills, spleen, lung, ovary, liver, heart, muscle and secretory field from one female and testis and secretory field from one male, and assembled the transcripts with and without the reference genome independently. Tophat and cufflinks 2.1.1^44^ were used for the with-reference assembly. For the without-reference assembly, we used Trinity 2.4.0 and PASA 2.2.0^45,46^. All gene evidence concluded by exonerate, Genewise, AUGUSTUS,GeneMark-ES, Tophat and cufflinks, and Trinity and PASA were collected and transferred to EVidenceModeler1.1.1^47^ to screen for high quality gene models that are supported by all lines of evidence. Those high-quality gene models were then used to train AUGUSTUS for improved gene predictions specifically for arapaima. Finally, the trained AUGUSTUS was run again with all previously obtained gene evidence as input, to predict the final set of gene models for arapaima. To access the quality of the annotation result, we mapped the resulting sequences to Pfam^48^ using InterProScan 5^49^ to investigate the portion of results that can be annotated with a known protein domain. We also used BUSCO^18^ based on the vertebrata odb9 database to access the annotation completeness. To assign gene symbols, we compared those gene sequences to the UniProt database (www.uniprot.org/e) using BLAST with a criterion e-value of 1E-5 (blastp2.2.28+^50^), and took the symbol of the best hit.

### Orthology inference

To form a protein pool, we unified 338,336 protein sequences from 12 species *L*. *oculatus* (spotted gar), *O*. *latipes* (Japanese medaka), *X*. *maculatus* (southern platyfish), *O*. *niloticus* (Nile tilapia), *G*. *aculeatus* (three-spined stickleback), *T*. *rubripes* (torafugu), *G*. *morhua* (Atlantic cod), *D*. *rerio* (zebrafish), *L*. *chalumnae* (coelacanth) (Ensembl release 87), *A*. *anguilla* (European eel) (http://www.zfgenomics.com/sub/eel), *S*. *formosus* (Asian bonytongue) and arapaima (our annotation). We then used blastp2.2.28+^50^ to compare the pool to itself. Based on the resulting raw score, we calculated an H-score as are^51^ of sequence distance for each pair of queries and hits, and clustered the proteins into groups by Hcluster_sg^52^. For each group, we used TreeBeST 0.5.1^53^ to build a gene tree guided by NCBI taxonomy phylogeny relationships (https://www.ncbi.nlm.nih.gov/taxonomy) and to infer orthology relationships. Ortholog relationships were then categorized as one-to-one, one-to-many or many-to-many using an in-house Perl (https://www.perl.org) script.

### Phylogenetic analysis

One-to-one orthologs across the 12 species were aligned as protein sequences using MUSCLE 3.8.31^54^. These alignments were filtered by trimAl^55^ with the parameters “-gt 0.8 –st 0.001 –cons 60” and then concatenated into a huge alignment. Based on the concatenated alignment, we reconstructed the phylogenomic tree for the 12 species using RAxML 8.2.9^56^ with PROTGAMMAAUTO parameter to select the optimal amino acid substitution model with coelacanth set as the outgroup and 100 bootstraps to test robustness. The fourfold degenerate site alignment corresponding to the concatenated protein alignment was then also subjected to RAxML through option “-f e” under the general time reversible (GTR) model to optimize the branch lengths for the phylogenomic tree. To confirm the topology of this tree, we also repeated the phylogenetic reconstruction using MrBayes 3.2.6, during which two simultaneous, independent runs were performed for 100,000 iterations of a Markov Chain Monte Carlo algorithm, with six simultaneous chains and sampling trees every 200 generations, resulting in 500 trees. The first 100 trees were “burned in”, and the average standard deviation of split frequencies remained ≤ 0.01 after the burn-in threshold. Divergence times along the phylogenomic tree was then estimated under relaxed clocks as implemented in MCMCTree^57^, with the CDS sequence alignment corresponding to the concatenated protein alignment used as input, including four fossil records: *O*. *latipes*–*T*. *nigroviridis* (~96.9–150.9 million years ago (Mya)), *D*. *rerio*–*G*. *aculeatus* (~149.85–165.2 Mya)^58^, *A*. *gigas*–*S*. *formosus* (~140–200 Mya)^23,24^ and sarcopterygians–actinopterygians (~400–500 Mya)^23^ as constraint. The MCMC process was run for 1,500,000 steps and sampled every 150 steps.

### Expansion and contraction of gene families

Gene family expansion and contraction was analyzed with the program CAFE 3.0^59^, in a maximum likelihood framework, using as input the gene group (family) size result from Hcluster_sg and the phylogenic tree from phylogenetic analysis. We instructed the program to search for the maximum likelihood value of birth and death rate (λ) following parameters “-p 0.01 -r 10000”. This means using 10,000 Monte Carlo random samplings to determine the probability of a gene family with the observed sizes and its birth and death rate, and then only of gene family with probabilities less than 0.01 to report the birth and death rate (λ). Before inputting to the CAFE program, gene families with no homology in the SWISS-PROT database or with multiple functional annotations were removed.

### Analysis of odorant receptor genes

ORs were identified from the genomes of torafugu, Japanese medaka, grass carp, Wuchang bream, channel catfish, Mexican tetra, zebrafish, arapaima, Asian bonytongue, European eel, spotted gar, coelacanth, elephant shark, green anole and chicken (http://www.ensembl.org)^60–62^. The method to identify and classify odorant receptor (OR) genes was adapted from Niimura^26^. First, TBLASTN was conducted to search whole genome sequences. As query, we extracted 1593 functional odorant receptor genes from 11 non-mammalian chordate species^27^. Because multiple queries will map to the same genome region, for each non-overlap hit region we took the query with the highest raw score as the best query. We then extended the non-overlapping hit regions on both sides and compared them to their best queries using Genewise. Coding sequences were extracted and extended to the start (ATG) and stop codons. Finally, we translated the coding sequences and kept those with more than 250 amino acids and no premature stop codon as final functional OR-potential genes. To assign them into groups (α–λ), we blasted them to a database containing the sequences of 1,593 functional ORs and 59 Non-ORs^50^. After discarding results with more than 40% identical matches, we assigned each query to the group with the best hit.

### dN/dS analysis

The dN/dS analysis was performed mainly with the CodeML program from the PAML package^57^. First, for the 12,929 one-to-one orthologs between arapaima and Asian arowana, pairwise dN/dS values were calculated and those with dN/dS > 1 were kept as positively selected gene candidates. Second, for the 5,882 one-to-one orthologs among arapaima, Asian arowana and European eel, the arapaima-European eel and Asian arowana-European eel pairwise dN/dS values were calculated, respectively. We then ranked those orthologs according to value *|log*_*2*_*(ω*_*1*_*/ω*_*2*_*)|* (ω_1_ refers to arapaima-European eel pairwise dN/dS value and ω_2_ refers to Asian arowana-European eel pairwise dN/dS value, and kept the top 5% that they are showed significant difference between arapaima and Asian arowana lineages in dN/dS value. Third, for the 3,128 one-to-one orthologs found in at least in 8 of the 12 species (arapaima, Asian arowana and European eel included), we screened arapaima, Asian arowana, European eel and the two most recent common ancestors (MRCA) lineages, respectively, for positively selected gene candidates, using CodeML in the branch-site model (model = 2 & NSsites = 2), with model A (fix_omega = 0) compared with the null model (fix_omega = 1 & omega = 1). For each analysis, all multiple sequence alignments were completed using MUSCLE in protein model, followed by pal2nal.pl^63^ for the protein to CDS sequence translation, and then filtered by Gblocks 0.91b^64^ in CDS model (−t = c), with alignment length <150 results excluded. All statistical analyses (likelihood ratio test, false discovery rate, value calculation and list cutting off) were carried out in R (https://www.r-project.org/).

### Identification of proteins with arapaima-unique residues

Sequences were aligned and gap-removed for 6052 homologs (orthologs and paralogs) retrieved from all 12 fish species. Alignments were analyzed by an in-house Perl script to identify and score the unique arapaima amino acid residues. The unique residue score was measured according to the variance of flanking residues across species:

$${\rm{S}}({\rm{p}})=\{\begin{array}{c}\sum _{{\rm{i}}=0}^{{\rm{p}}+25}{{\rm{V}}}_{{\rm{i}}}{(\frac{|{\rm{i}}-{\rm{p}}|}{10}+1)}^{-2},0\le {\rm{p}} < 25\\ \sum _{{\rm{i}}={\rm{p}}-25}^{{\rm{p}}+25}{{\rm{V}}}_{{\rm{i}}}{(\frac{|{\rm{i}}-{\rm{p}}|}{10}+1)}^{-2},25\le {\rm{p}} < l-25\\ \sum _{{\rm{i}}={\rm{p}}-25}^{{\rm{l}}}{{\rm{V}}}_{{\rm{i}}}{(\frac{|{\rm{i}}-{\rm{p}}|}{10}+1)}^{-2},{\rm{l}}-25\le {\rm{p}} < {\rm{l}}\end{array},$$in which p stands for the location of the unique arapaima amino acid residues in the alignment; S(p), the score; ${V}_{i}$, the number of different residues in No. i position of the alignment; l the alignment length, thus lower flanking residue variance leading to a higher score for unique residues. Finally, the score for the sequence was determined as a synthesis of its unique residue scores normalized to protein length: ${\rm{S}}={\sum }^{}{\rm{S}}({\rm{p}})/{\rm{l}}$, in which ${\sum }^{}{\rm{S}}({\rm{p}})$ stands for the score sum of the unique residues it harbors; l, the alignment length.

### GO enrichment analysis

Enrichment of GO terms for the genes that were selected in dN/dS analysis and identification of proteins with arapaima-unique residues was determined using the TopGO package from Bioconductor (http://www.bioconductor.org), which employs Fisher’s exact test and 2 × 2 contingency tables to check for significant over-representation of GO terms in one set compared with another set. GO categories with p < 0.05 were considered significantly enriched. Whole arapaima gene were used as background.

### Sex-specific sequences

To extract sex-specific scaffolds, at first, the male and female genomes were aligned one versus the other using blat (male versus female and vice versa). Then blat hits were filtered and hits having a match length greater than 100 bp, a mismatch rate (match length divided by mismatch length) lower than 3% and coverage (match length divided by query length) greater than 10% were retained.

An R script was used to select all contigs from the first genome having no hits from the second genome covering them. This was performed for both genomes. The corresponding contigs were considered as male or female genome specific.

Male-linked RAD-tags were blasted to the sex-specific scaffolds and the rest of the reference genomes (both male and female), and only the best hit for each query were kept. To find possible duplications, scaffolds from the male specific collection were blasted to the remainder of the male reference genome.

### Transcriptome analysis

Total RNA was isolated using TRIzol Reagent (Thermo Fisher Scientific, Waltham, USA) according to the supplier’s recommendation. RNA from eyes (RIN 8), gills, spleen (RIN 7.8), lung (RIN 7.9), ovary (RIN 7.1), liver (RIN 7.7), heart (RIN 7.6), muscle (RIN 7.2) and secretory field (RIN 8.5) from one female and testis (RIN 8.1) as well as secretory field (RIN 7.5) from one male were obtained from the broodstock of the Leibniz-Institute of Freshwater Ecology and Inland Fisheries (IGB). The settlings were imported from Neotropical Fauna E.I.R.L (Iquitos, Peru) in 2013 (CITES 1054487) and raised at the facilities of the IGB. Maturing fish were sacrificed by state-of-the-art humane killing (American Veterinary Medical Association, Canadian Council of Animal Care in Science). The experiments were carried out in accordance with the European Directive 2010/63/EU and German national legislation. RNA-Seq reads were used as transcriptomic evidence for genome annotation and sex-biased expression analysis. Custom sequencing (BGI, Shenzen, China) of TruSeq libraries generated 25–30 million 100 bp paired end reads for each sample on the Illumina Hiseq4000 platform.

### Differential gene expression

Genes were aligned to the arapaima transcripts using Bowtie2^65^ with default settings. Differentially expressed genes were detected using the Bioconductor package DESeq2^66^. A gene was considered to be expressed, if at least ten reads were detected. A gene was considered to be differentially expressed for the comparison ovary vs. testis, if a gene was expressed with read counts >500 in one gonad and unexpressed in the other. For the comparison male vs. female secretory organ a gene was required to have an expression value of at least 500 in male or female and a log2FC > 4. These highly stringent criteria were chosen to account for the fact that there were no replicates. For functional enrichment analysis the web tool DAVID (https://david.ncifcrf.gov/) has been used with human as reference. Venn diagrams were drawn using the online tool Venny (http://bioinfogp.cnb.csic.es/tools/venny/).

## Supplementary information

Supplementary information

Dataset 1
