# Supplementary material for: The genome of the arapaima (Arapaima gigas) provides insights into gigantism, fast growth and chromosomal sex determination system
Source: Sci Rep. 2019 Mar 28;9:5293. doi: 10.1038/s41598-019-41457-x (PMC6439221; doi:10.1038/s41598-019-41457-x)
Supplement: Supplementary file 1 — Supplementary information [file 41598_2019_41457_MOESM1_ESM.pdf]

The genome of the arapaima (*Arapaima gigas*) provides insights into gigantism, fast growth and chromosomal sex determination system

Du Kang[1,2,3], Sven Wuertz[4], Mateus Adolfi[1], Susanne Kneitz[1], Matthias Stöck[4], Marcos Oliveira[1,5], Rafael Nóbrega[5], Jenny Ormanns[1], Werner Kloas[4], Romain Feron[6], Christophe Klopp[7], Hugues Parrinello[8], Laurent Journot[8], Shunping He[2], John Postlethwait[9], Axel Meyer[10], Yann Guiguen[6], Manfred Schartl[1,11,12,*]

[*]Corresponding author

[1] University of Wuerzburg, Physiological Chemistry, Biocenter, 97074 Wuerzburg, Germany (dukang1117@foxmail.com; mateusadolfi@gmail.com, phch1@biozentrum.uni-wuerzburg.de)

[2] Key Laboratory of Aquatic Biodiversity and Conservation of the Chinese Academy of Sciences, Institute of Hydrobiology, Chinese Academy of Sciences, Wuhan, Hubei 430072, China

[3] University of Chinese Academy of Sciences, Beijing 100049, China

[4] Leibniz-Institute of Freshwater Ecology and Inland Fisheries, IGB, Müggelseedamm 301, D-12587 Berlin, Germany (wuertz@igb-berlin.de, matthias.stoeck@igb-berlin.de)

[5] Reproductive and Molecular Biology Group, Departament of Morphology, Institute of Biosciences, UNESP, Botucatu, Brazil (oliveira@zootecnista.com.br, biorhn@yahoo.com.br)

[6] INRA, UR1037 LPGP, Fish Physiology and Genomics, F-35042 Rennes, France (yann.guiguen@inra.fr)

[7] MIAT INRA Toulouse, CS 52627, 31326 Castanet-Tolosan, France (christophe.klopp@inra.fr)

[8] Montpellier GenomiX (MGX), c/o Institut de Génomique Fonctionnelle, 141 rue de la cardonille, 34094 Montpellier Cedex 05, France (Hugues.Parrinello@mgx.cnrs.fr)

[9] Institute of Neuroscience, University of Oregon, Eugene, Oregon, OR 97401, USA.

[10] Lehrstuhl für Zoologie und Evolutionsbiologie, Department of Biology, University of Konstanz, Universitätstraße 10, 78457 Konstanz, Germany

[11] Comprehensive Cancer Center Mainfranken, University Hospital, 97080 Würzburg, Germany

[12] Hagler Institute for Advanced Study and Department of Biology, Texas A&M University, College Station, Texas 77843, USA

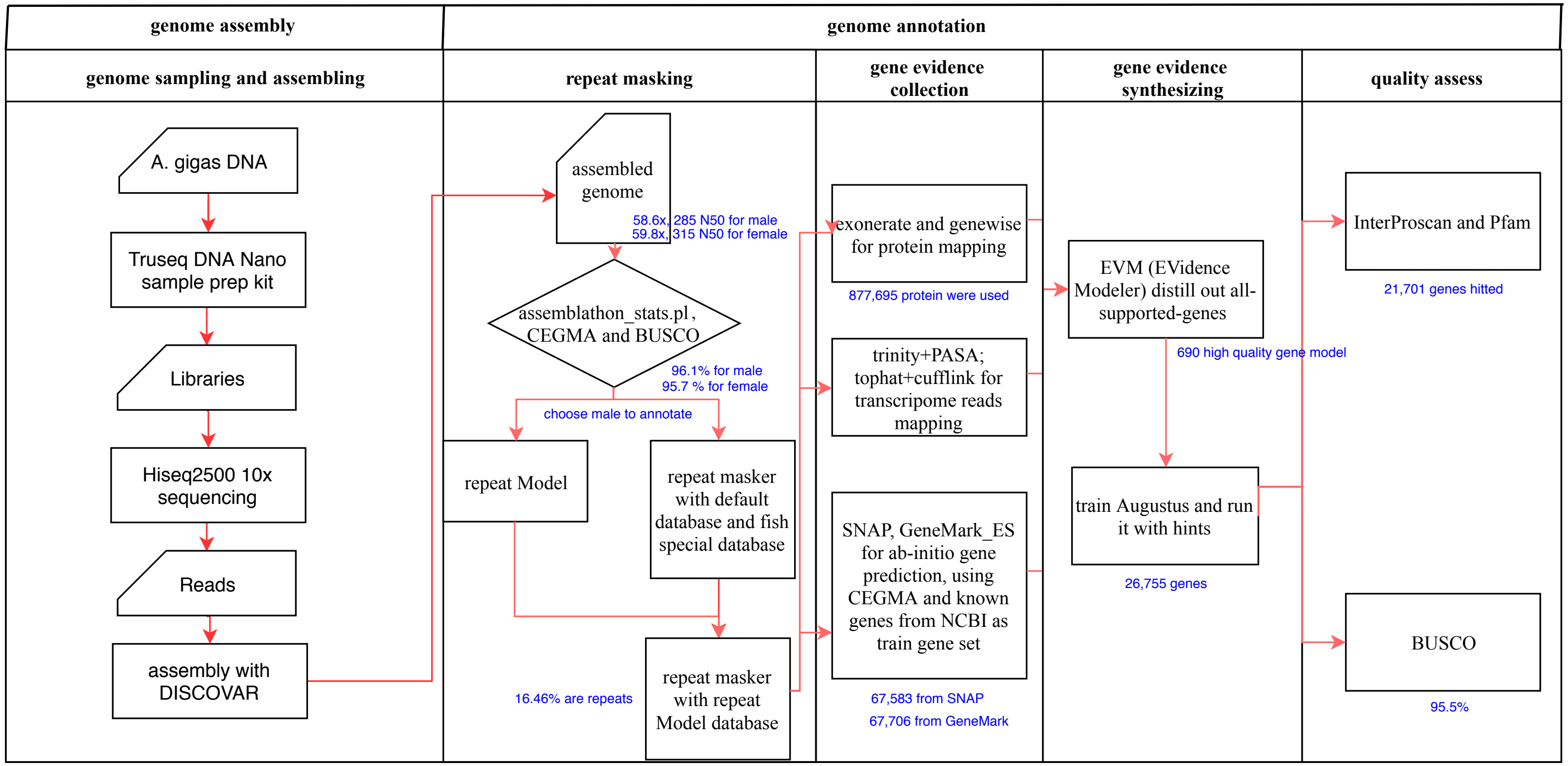

Figure S1. The flow chart showing the process of genome assembly and annotation.

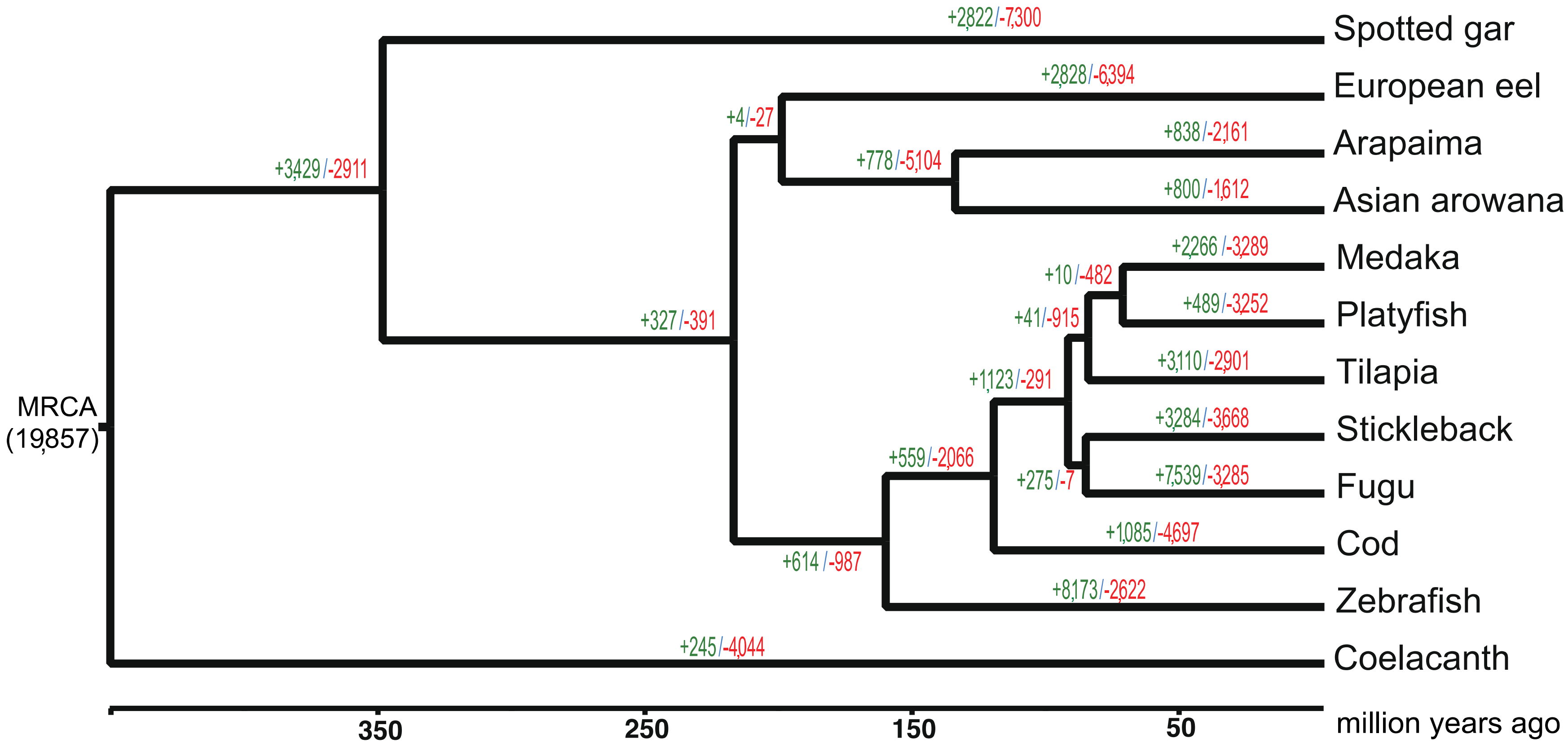

Figure S2. Statistical analysis of contraction and expansion of gene clusters.

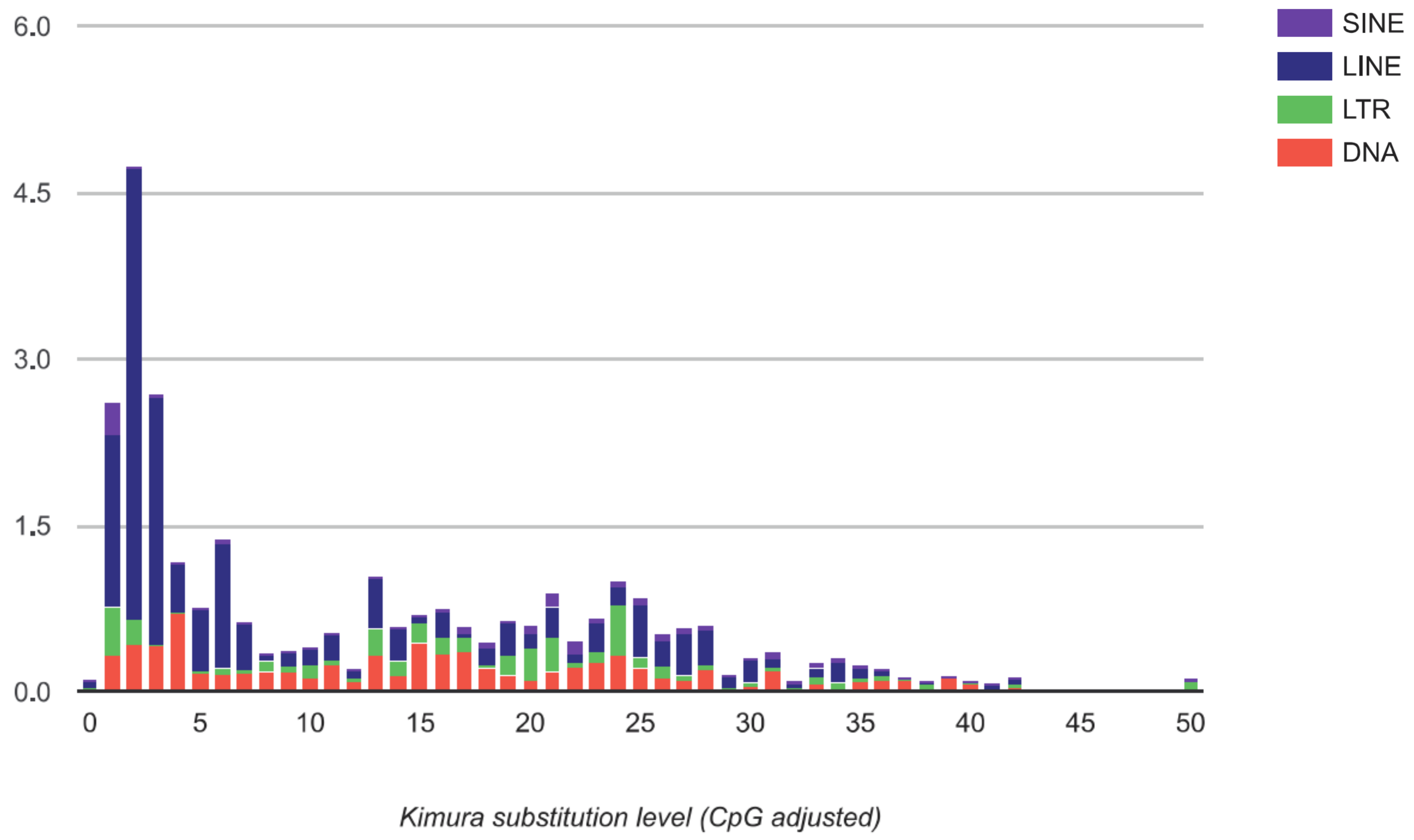

Figure S3. Interspersed repeat landscape for male-specific scaffolds of arapaima. The interspersed repeat landscape, revealing the copy-divergence analysis of TE classes, based on Kimura distances. Percentages of TEs in genomes (Y-axis) are clustered based on their Kimura values (X-axis; K-values from 0 to 50; arbitrary values). Older copies are located on the right side of the graphs while rather recent copies are located on the left side.

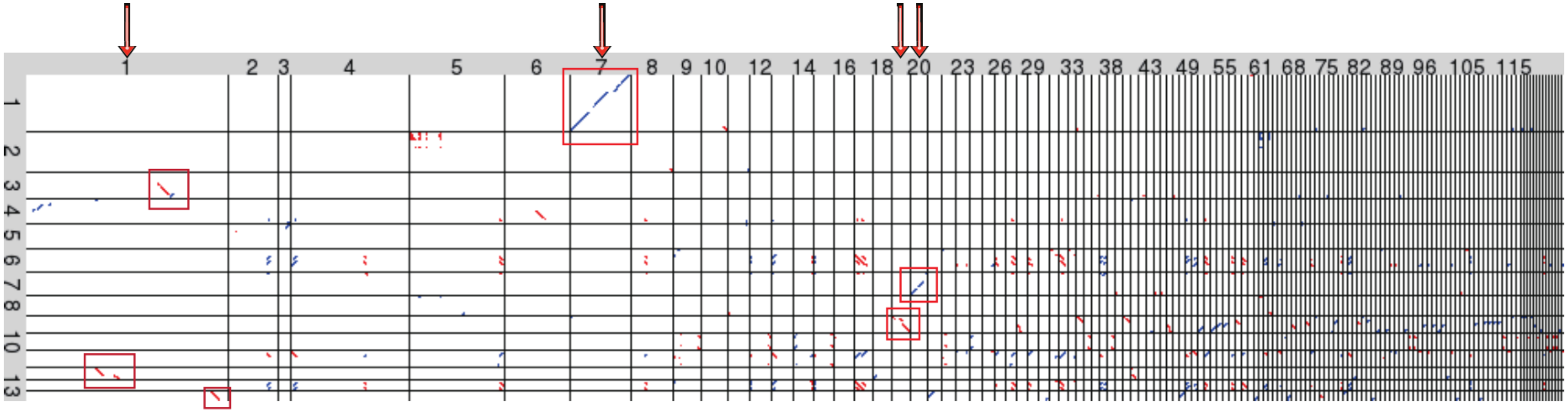

Figure S4. Dotplot diagram from LAST revealing the alignments between male-specific scaffolds (vertical) and the rest of the male reference genome (horizontal). The red frame indicates continuous alignment between query and hit. Scaffolds on the horizontal axis with red arrays (1, 7, 19, 20) represent scaffolds harboring regions that are potentially paralogous to male-specific scaffolds.

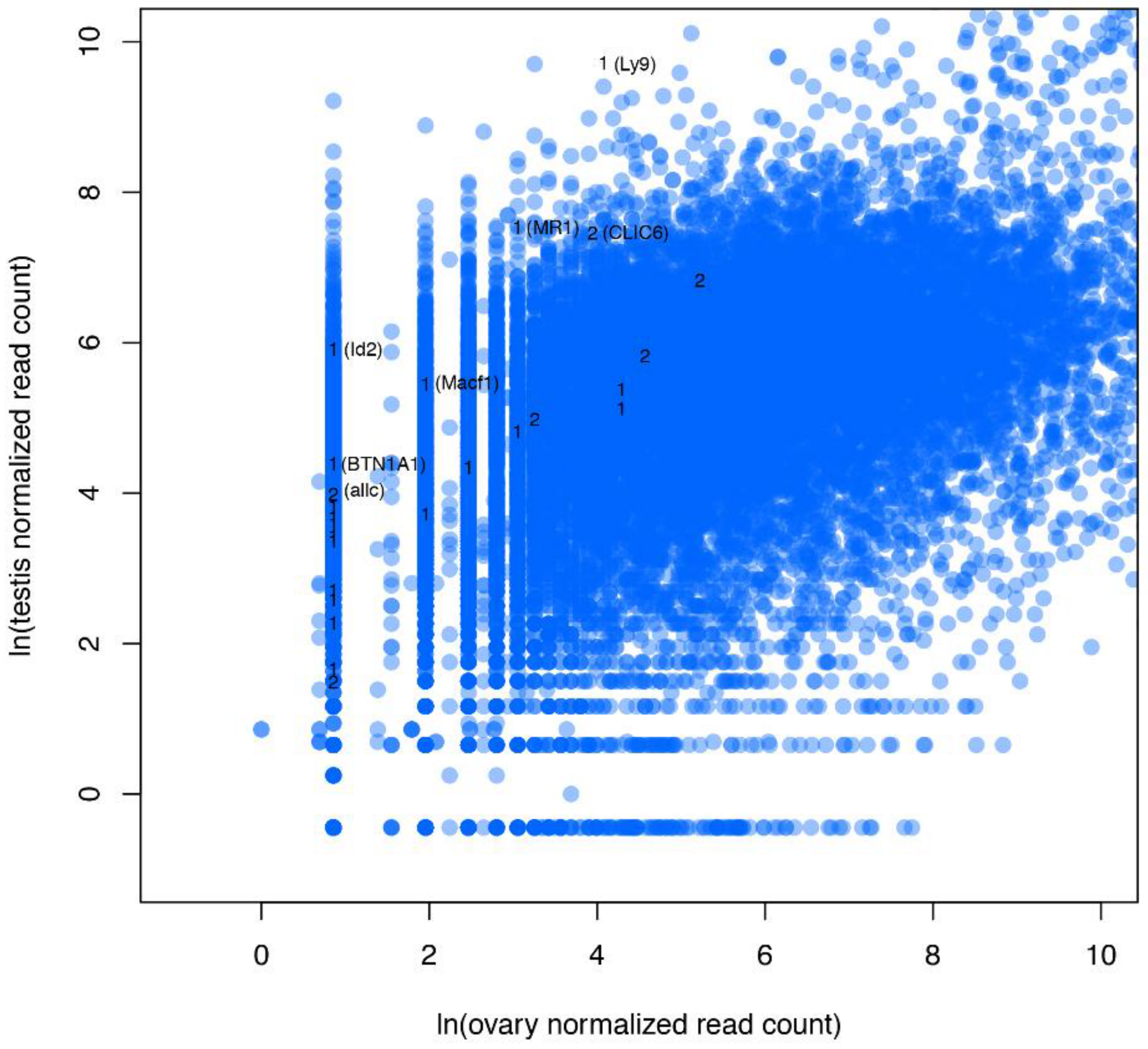

Figure S5. Dotplot diagram revealing the natural log value of normalized read counts in testis (horizontal axis) and in ovary (vertical axis). Genes that are located in male-related scaffolds are marked in black numbers ('1' refers to genes located in male-specific scaffolds and '2' refers to the paralogous autosomal region of male-specific scaffolds).

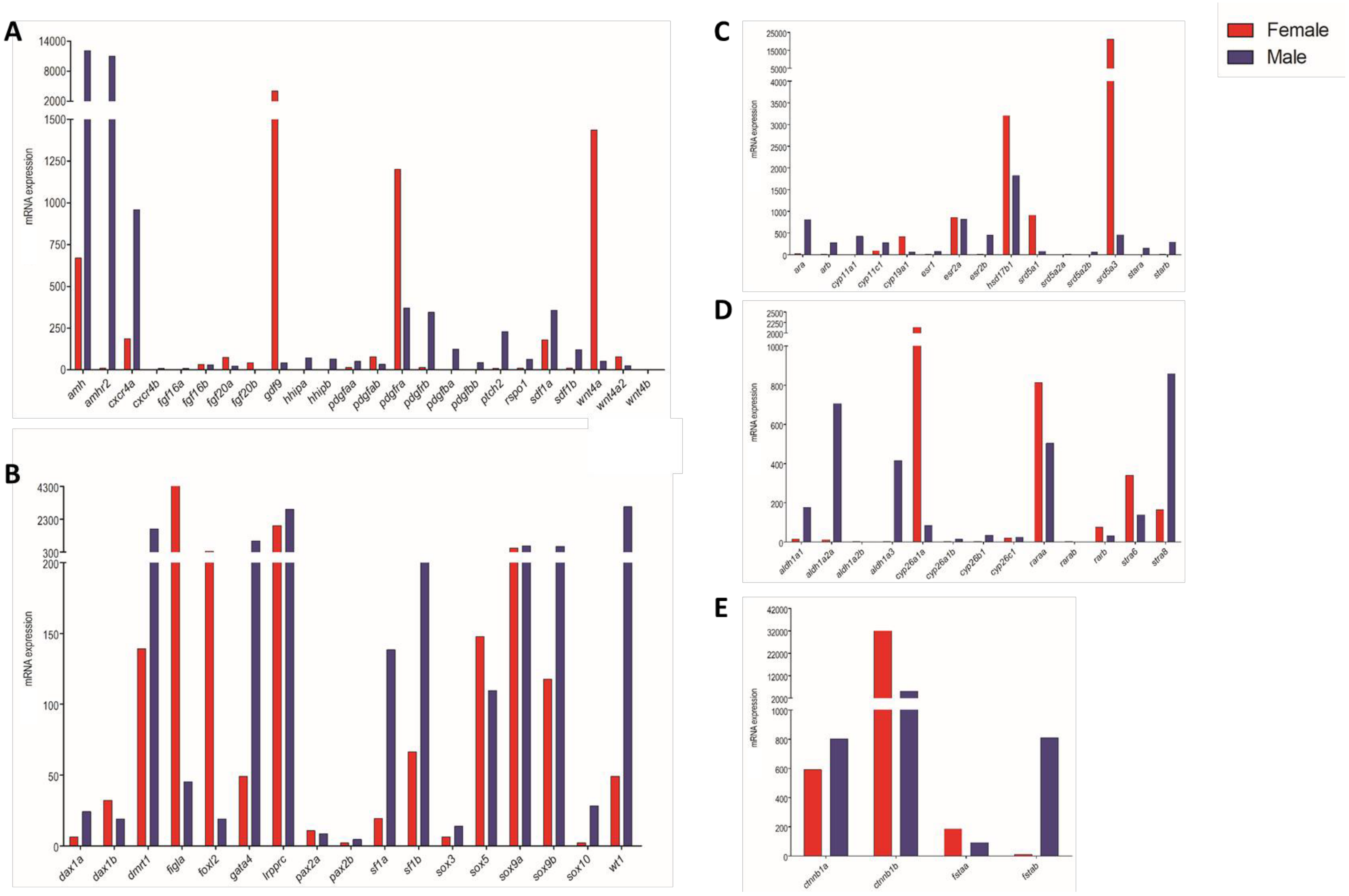

Figure S6. Expression levels of genes related to sex differentiation and gametogenesis in arapaima gonads. The expression levels are presented as normalized read counts calculated using DESeq2. A) Growth factors and receptors. B) Transcription factors. C) Steroidogenic enzymes and receptors. D) Meiosis related genes. E) Others.

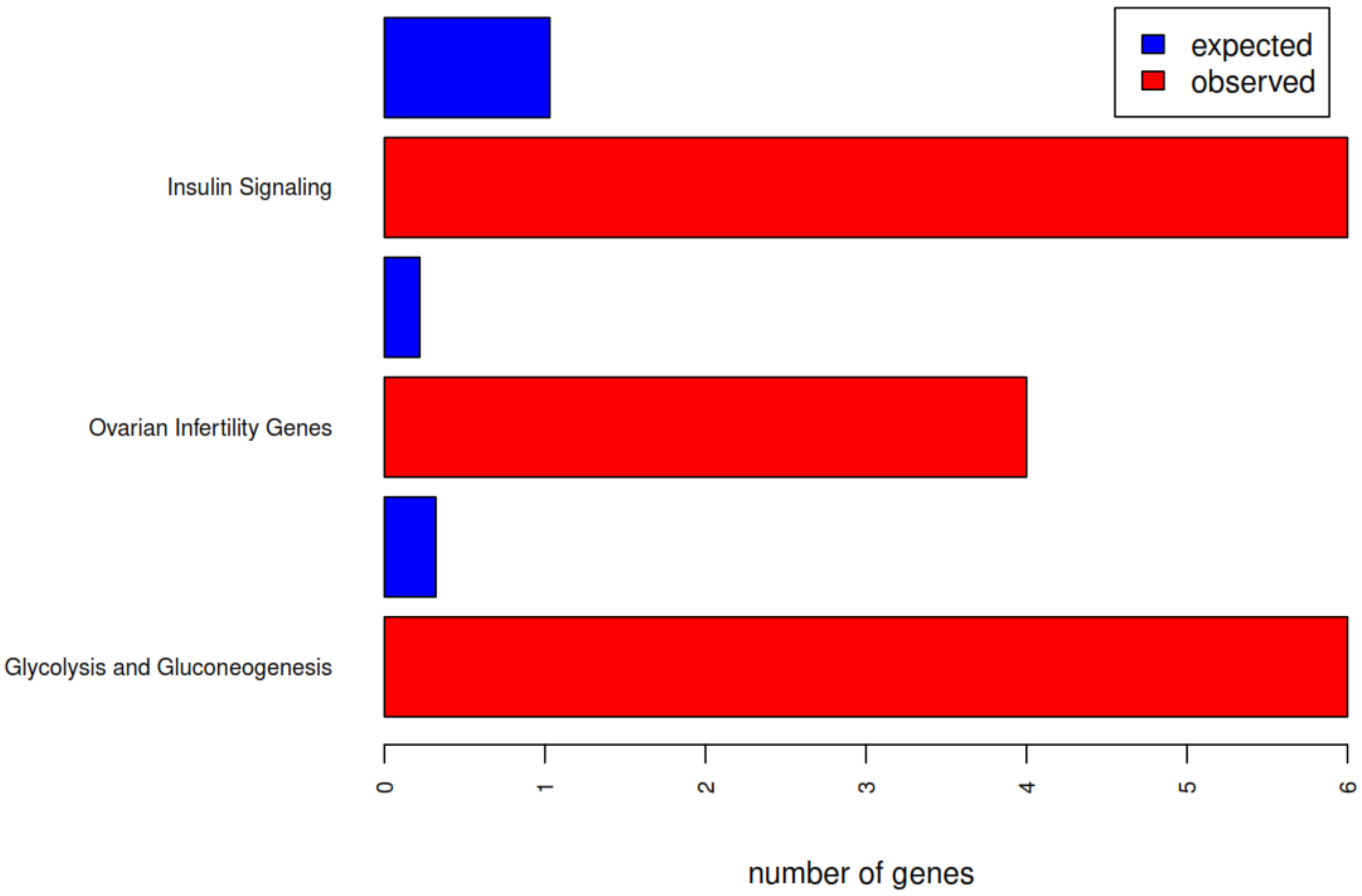

Figure S7. Barplot of the expected and observed number of genes in significantly enriched wiki pathways (adj.pval<0.01) for the comparison between male and female secretory organ. Blue indicates the number of expected genes; red indicates the number of differentially expressed genes.
